# Pazopanib as maintenance therapy in pediatric ewing sarcoma: a case series of six patients

**DOI:** 10.1186/s40348-025-00213-0

**Published:** 2025-12-03

**Authors:** Nihan Bayram, Murat Elli, Yontem Yaman, Isık Odaman Al, Kursat Ozdilli, Dilek Unal, Harzem Ozger, Sema Anak

**Affiliations:** 1 Department of Pediatric Hematology and Oncology, Bezmi Alem University, Istanbul, Turkey; 2 Department of Pediatric Hematology and Oncology, Istanbul Medipol University, Istanbul, Turkey; 3 Department of Medical Biology, Istanbul Medipol University, Istanbul, Turkey; 4 Department of Radiation Oncology, Istanbul Medipol University, Istanbul, Turkey; 5 Department of Orthopedic Surgery, Acıbadem University Hospital, Istanbul, Turkey

## Abstract

**Introduction:**

Ewing sarcoma (ES) is an aggressive pediatric bone and soft tissue malignancy. Despite advances in multimodal therapy, outcomes remain suboptimal for patients with high-risk features such as large tumor volume, poor histologic response, disseminated disease, or relapse. Maintenance strategies have gained interest in pediatric sarcomas, but evidence in ES is limited. Pazopanib, an oral multi-targeted tyrosine kinase inhibitor, has shown activity in adult soft tissue sarcomas; however, data on its use as maintenance therapy in pediatric ES are scarce.

**Methods:**

We retrospectively analyzed six pediatric ES patients who received pazopanib as maintenance therapy following consolidation treatment or, when applicable, after high-dose chemotherapy with autologous stem cell transplantation. Indications included poor-risk localized disease, relapse, or stable residual disease after frontline therapy. Pazopanib dosing, duration, toxicity, and clinical outcomes were reviewed.

**Results:**

Pazopanib was administered at doses of 400–800 mg in five patients and 200 mg twice daily in one patient. Hair depigmentation occurred in all patients, and one patient developed reversible hepatotoxicity requiring temporary interruption and dose reduction. At a median follow-up of 47 months (range, 18–53 months), five patients remained in complete remission. One patient with disseminated disease at diagnosis developed intracranial relapse 18 months post-transplant while still receiving pazopanib.

**Conclusions:**

In this retrospective cohort, pazopanib was generally well tolerated and may have a potential role as maintenance therapy in selected high-risk pediatric ES patients. However, efficacy cannot be determined from this limited series, and prospective studies are needed to better define its clinical utility in ES.

## Introduction

Ewing sarcoma (ES) and primitive neuroectodermal tumors (PNET) are rare malignancies characterized by small round cells, typically arising in bone or soft tissue, and predominantly affecting children and young adults [1].

Patients with ES are currently treated with multimodal therapy consisting of induction chemotherapy, local control, and risk-adapted consolidation, most commonly based on VDC/IE (vincristine, doxorubicin, ifosfamide, etoposide) or VIDE (vincristine, ifosfamide, doxorubicin, etoposide) protocols [2, 3]. Risk stratification integrates disease extent, tumor burden, and histological response. At diagnosis, patients are categorized as having localized disease, lung-only metastases, or disseminated disease (including extrapulmonary metastases or bone marrow involvement). Among patients with localized disease, those with a favorable histological response and smaller tumor volume are considered standard-risk, whereas those with adverse features—including poor response (>10% viable tumor) or large tumor volume (>200 mL)—are classified as high-risk [4].

Five-year event-free survival (EFS) is approximately 60–70% in patients with localized disease but declines to 23–26% in those with lung-only metastases [2, 5–7]. Prognosis is particularly poor in very high-risk cases, including disseminated disease, where long-term survival may be as low as ~ 20% despite intensive multimodal therapy. These outcomes underscore the need for novel strategies to improve survival in high-risk ES [7].

First-line treatment typically consists of multi-agent chemotherapy, most commonly the VDC/IE or VIDE regimen, as established in the EURO-E.W.I.N.G. 99 [8, 9] and COG AEWS0031 trials [10]. In the relapsed setting, although multiple salvage regimens have been explored, no universally accepted standard treatment exists [3, 5, 6].

Maintenance therapy has emerged as a strategy to suppress minimal residual disease (MRD) following intensive treatment, with promising evidence in other pediatric sarcomas such as rhabdomyosarcoma. By targeting residual tumor cells not detectable on conventional imaging, maintenance approaches aim to prolong remission and delay relapse [11].

Pazopanib, an oral multi-targeted tyrosine kinase inhibitor, has demonstrated efficacy in prolonging progression-free survival in patients with advanced soft tissue sarcomas [12]. However, its role in ES, particularly in pediatric patients, remains unclear, and evidence supporting its use as maintenance therapy is limited to isolated reports [13, 14].

Herein, we describe six pediatric ES cases in which pazopanib was incorporated as maintenance therapy following consolidation or salvage treatment in carefully selected high-risk patients.

## Patients and treatment

This retrospective analysis included patients diagnosed with Ewing sarcoma and treated at Istanbul Medipol University Pediatric Oncology Department between 2020 and 2023. Maintenance therapy with pazopanib was considered for patients classified as high-risk, including those with axial tumor location, incomplete surgical resection, relapse or other adverse prognostic features. Informed consent was obtained from all patients, and the study was approved by the institutional ethics committee.

Since there is no nationally established Ewing sarcoma protocol in Turkey, our induction chemotherapy for newly diagnosed patients was adapted from the Euro-Ewing protocols: while the Euro-Ewing 2012 protocol recommends six cycles of VIDE (vincristine 1.5 mg/m^2^ day1, ifosfamide 3 g/m^2^/d day 1–3, doxorubicin 20mg/m^2^/d day 1–3, etoposide 150mg/m^2^/d d1-3) as induction, we administered four cycles of VIDE followed by two cycles of IE (ifosfamide 3 g/m^2^/d d 1–3, etoposide 150mg/m^2^/d d 1–3) concurrently with radiotherapy. This approach minimized interruptions in systemic therapy while remaining aligned with Euro-Ewing treatment principles.

After the concurrent IE cycles administered with radiotherapy, patients underwent surgical resection, and subsequently, consolidation chemotherapy was administered according to their risk group, following the principles of the Euro-Ewing protocols. Patients with localized disease who remained classified as poor-risk following induction therapy and local control received high-dose chemotherapy with busulfan and melphalan, followed by autologous stem cell transplantation as consolidation therapy. Busulfan was initiated at 0.8–1.2 mg/kg with pharmacokinetic-guided adjustments over 16 doses, and melphalan was administered at 140 mg/m² on day − 2. In Case 1, however, a treosulfan–melphalan regimen (TreoMel) was used instead of BuMel because the patient relapsed with two pulmonary nodules and was scheduled to receive pulmonary radiotherapy. To minimize busulfan-associated pulmonary toxicity in the context of planned thoracic irradiation, treosulfan was administered at 14 g/m²/day on days − 6 to − 4, followed by melphalan 140 mg/m².

Remission status was assessed using MRI and CT, with PET-CT performed when clinically indicated. For patients treated with curative intent, pazopanib maintenance was initiated only in the absence of radiologic evidence of active disease. In contrast, pazopanib was introduced in the setting of radiologically stable residual disease for the patient with disseminated disease at diagnosis, who also underwent incomplete surgical resection.

Patient monitoring during pazopanib therapy was conducted at structured intervals: laboratory evaluations were performed twice weekly during the initial two weeks, weekly for the subsequent eight weeks, and monthly thereafter, while blood pressure was monitored daily by the patient at home and recorded for review during scheduled visits.

Pazopanib was initiated at 50% of the target dose and increased after two weeks, with maintenance dosing adjusted according to body weight (400–800 mg/day). Dose modifications were applied based on toxicity, and recurrence of symptoms or laboratory abnormalities was recorded after drug discontinuation.

Relapsed and refractory patients were treated with individualized salvage regimens. Pazopanib was initiated either after achieving complete remission following salvage therapy or, in the setting of stable residual disease.

## Case report 1

A 15-year-old girl presented with a swelling on her back and was diagnosed with ES localized to the scapula. Staging workup revealed no evidence of metastatic disease. She received four cycles of VIDE (vincristine, ifosfamide, doxorubicin, etoposide), followed by two cycles of IE (ifosfamide, etoposide) administered concurrently with radiotherapy, after which she underwent surgical resection and was classified as having localized, good-risk disease. Subsequently, she received eight cycles of consolidation chemotherapy alternating between VAI (vincristine 1.5mg/m^2^ day 1, actinomycin D 0.75mg/m^2^ day 1–2, ifosfamide 3 g/m^2^/d d 1–2) and VAC (vincristine 1.5mg/m^2^ day 1, actinomycin D 0.75mg/m^2^/d d 1–2, cyclophosphamide 1500mg/m^2^ day 1) regimens.

Ten months after the completion of frontline therapy, she experienced a local recurrence accompanied by two pulmonary nodules. The lung lesions were surgically resected, and histopathological examination confirmed metastatic ES. Salvage therapy was initiated with six cycles of VIT (vincristine 1.5mg/m^2^ day 1, irinotecan 50mg/m^2^ day 1–5, temozolomide 150mg/m^2^ day 1–5), followed by high-dose chemotherapy with treosulfan and melphalan, and autologous stem cell transplantation. Post-transplantation, she received pulmonary radiotherapy and subsequently maintenance therapy with pazopanib at a dose of 400 mg twice daily for one year. She remains in complete remission 18 months following transplantation.

## Case report 2

A 9-year-old girl presented with urinary and fecal incontinence and was diagnosed with ES involving the sacrum. Staging workup revealed no evidence of metastatic disease. She received four cycles of VIDE, followed by two cycles of IE administered concurrently with radiotherapy, after which she underwent surgical resection. Surgical resection was incomplete due to the high risk of worsening incontinence, and she was therefore classified as having localized, poor-risk disease. Subsequently, she received one cycle of VAI chemotherapy, followed by high-dose chemotherapy with busulfan and melphalan, and autologous stem cell transplantation.

Post-transplantation, she received maintenance therapy with pazopanib (200 mg twice daily) for one year. She remains in complete remission 45 months after transplantation.

## Case report 3

A 10-year-old girl presented with urinary retention and a palpable abdominal mass. She was diagnosed with pelvic ES originating from the ileum, and staging imaging revealed pulmonary metastases. She received four cycles of VIDE, followed by two cycles of IE administered concurrently with radiotherapy, after which she underwent surgical resection. Subsequently, she received one cycle of VAI chemotherapy, followed by high-dose chemotherapy with busulfan and melphalan, and autologous stem cell transplantation.

Post-transplantation, she received maintenance therapy with pazopanib (600 mg daily) for one year. She remains in complete remission 53 months after transplantation.

## Case report 4

A 15-year-old girl presented with a very large mass (380 mL, exceeding the > 200 mL high-risk threshold) in the right leg and was diagnosed with ES. Staging workup revealed no evidence of metastatic disease. She received four cycles of VIDE, followed by two cycles of IE administered concurrently with radiotherapy, after which she underwent surgical resection. She subsequently received eight cycles of consolidation chemotherapy alternating between VAI and IE regimens.

Following chemotherapy, maintenance treatment with pazopanib was initiated at a dose of 400 mg twice daily. During the third month of therapy, she developed hepatotoxicity, characterized by an elevation of liver transaminases up to five times the upper limit of normal, prompting temporary discontinuation of the drug for three weeks. Once liver function normalized, treatment was resumed at a reduced dose of 200 mg twice daily, with no further toxicity observed thereafter. Maintenance therapy was completed over a total duration of one year. She remains in complete remission 52 months after completion of therapy.

## Case report 5

A 13-year-old girl presented with a palpable very large abdominal mass (290 mL, exceeding the > 200 mL high-risk threshold) and was diagnosed with pelvic ES originating from the ileum. Staging work-up revealed no evidence of metastatic disease. She received four cycles of VIDE, followed by two cycles of IE administered concurrently with radiotherapy, after which she underwent surgery. She subsequently received eight cycles of consolidation chemotherapy alternating between VAI and IE regimens.

Following the completion of chemotherapy, maintenance treatment with pazopanib was initiated at a dose of 400 mg twice daily and continued for one year. She has remained in complete remission for 43 months following the end of therapy.

## Case report 6

An 8-year-old boy presented with a palpable abdominal mass and was diagnosed with pelvic Ewing sarcoma originating from the ileum. At the time of diagnosis, imaging and bone marrow evaluation revealed pulmonary metastases and bone marrow involvement. He received six cycles of VIDE. After the fourth cycle, surgical resectability was evaluated; however, after the sixth cycle, surgical intervention was considered not feasible due to the extensive tumor involvement and the high risk of morbidity associated with resection, resulting in the decision to proceed without surgical resection, leading to incomplete local control.

Subsequently, he received two cycles of IE administered concurrently with definitive radiotherapy to the primary site. He then received one cycle of VAI chemotherapy, followed by high-dose chemotherapy with busulfan and melphalan, and autologous stem cell transplantation.

Given the presence of residual but radiologically stable disease following transplant, maintenance therapy with pazopanib (200 mg twice daily) was initiated on day 30 post-transplant.

Eighteen months after transplantation, while still receiving pazopanib, the patient experienced disease relapse with an intracranial mass.

## Results

Six pediatric patients with ES who received pazopanib as maintenance were included in this case series. At diagnosis, four patients had localized disease, while one had pulmonary-only metastases and one had disseminated disease. All patients received multi-agent chemotherapy and local treatment consisting of surgery and/or radiotherapy. Following induction and local therapy, four patients with localized poor-risk disease underwent high-dose chemotherapy followed by autologous stem cell transplantation (HDCT + ASCT), whereas two patients with standard-risk localized disease completed treatment without transplantation.

Pazopanib was initiated as maintenance with the rationale of targeting minimal residual disease (MRD) and preventing relapse. Four patients received pazopanib as maintenance therapy during first remission, one patient received it following relapse, and one patient with disseminated disease at diagnosis received pazopanib after completion of frontline therapy while maintaining stable disease.

Pazopanib was administered at doses ranging from 400 to 800 mg per day, determined according to patient weight and age, following previously reported pediatric dosing strategies [10]. Notably, all patients developed hair depigmentation (white or gray hair), a previously reported but benign side effect of pazopanib. One patient required temporary treatment interruption for three weeks due to hepatotoxicity, after which liver function normalized and therapy was resumed at a reduced dose of 200 mg twice daily without further toxicity. We did not observe hypertension, another potential adverse effect of pazopanib, in any of the six patients.

Pazopanib was administered for one year in five patients, and for 18 months in one patient who was unable to undergo surgical resection. Five patients remain in complete remission at a median follow-up of 47 months (range: 18–53 months). One patient relapsed with an intracranial mass while still receiving pazopanib.

A summary of clinical characteristics and outcomes is provided in Table 1.

**Table 1:** Clinical characteristics, treatment details, and outcomes of pediatric ewing sarcoma patients receiving pazopanib

Patient	Age (y)	Sex	Stage at diagnosis	Rationale for Maintenance	Dose (mg/day)	Duration (months)	Follow-up, months
1	15	F	Localized	Relapse	800	12	CR, 18
2	9	F	Localized	Incomplete surgery	400	12	CR, 45
3	10	F	Pulmonary-only metastasis	Metastatic disease	600	12	CR, 53
4	15	F	Localized	>200 ml Tumor volume	800 400	12	CR, 52
5	13	F	Localized	> 200 ml Tumor volume	800	12	CR, 43
6	8	M	Disseminated	Dısseminated disease	400	18	Relapse (IC mass), 18

*Abbreviations: IC* Intracranial

## Discussion

Ewing sarcoma (ES) is an aggressive pediatric bone and soft tissue malignancy [1], and despite substantial therapeutic advances, outcomes remain suboptimal for patients with high-risk features. Prognosis is particularly poor in those with large tumor volume, poor histologic response, disseminated disease, or relapse [1, 2, 5, 7]. These unfavorable outcomes have prompted increasing interest in strategies aimed at reducing the risk of recurrence following intensive multimodal therapy, including high-dose chemotherapy and autologous stem cell transplantation (ASCT) [7].

Pazopanib is a multi-targeted tyrosine kinase inhibitor with anti-angiogenic properties that has been shown to improve progression-free survival in adults with advanced soft tissue sarcomas [12, 15]. Although it has not received regulatory approval for pediatric bone sarcomas, its potential activity in osteosarcoma (OS) and ES has been suggested mainly through retrospective series and case reports [13, 16–21]. However, robust prospective data remain limited [13, 22].

A recent retrospective study included 19 patients with bone sarcomas (8 with OS, 3 with ES). The clinical benefit rate was 68%, with a median progression-free survival of 5.45 months (95% CI: 2.7–7.7)^16^. Notable responses were observed in individual patients, including a 24-year-old with metastatic, chemotherapy-refractory ES who achieved a partial response lasting 12 weeks [17]. Clinical activity has also been reported in extra-osseous ES, with one patient showing temporary tumor shrinkage and another demonstrating a 70% reduction in a local lesion along with stabilization of liver metastases for two months [18, 19].

In pediatric populations, phase I and II trials have primarily evaluated tolerability and pharmacokinetics, with limited efficacy data. Dose-limiting toxicities included elevated lipase, amylase, ALT, proteinuria, and hypertension, and the maximum tolerated dose was 450 mg/m² for the tablet formulation. Although objective responses were infrequent, several patients achieved prolonged stable disease, suggesting a potential clinical benefit in selected patients [13]. Furthermore, a recent multicenter, real-world retrospective study of 19 pediatric and young adult patients with solid tumors, including bone and soft tissue sarcomas, reported disease stabilization in 52.6% at 8 weeks, with a 6-month disease control rate of 26.3% ^20^ Although earlier initiation of pazopanib appeared to correlate with improved short-term stabilization in that cohort, the small sample size limits the interpretability of this observation. A remarkable case involved a 17-year-old patient with metastatic ES who achieved sustained disease control on pazopanib monotherapy for 26 months [21].

Interest in maintenance therapy for sarcomas has increased following the positive results of the European rhabdomyosarcoma trial demonstrating the benefit of metronomic cyclophosphamide and vinorelbine [11]. In ES, the CWS-2002 protocol explored optional maintenance therapy in extraskeletal disease, but interpretation is limited by small sample size and potential selection bias [23]. Other maintenance strategies, including trofosfamide and targeted agents such as ganitumab, have shown inconsistent benefit [24, 25]. Overall, maintenance therapy in ES remains investigational and is not considered standard of care.

Tamura et al. reported the first pediatric case using pazopanib as maintenance therapy following ASCT. A 14-year-old boy with metastatic pelvic ES received tandem high-dose chemotherapy and radiotherapy after poor responses to initial and salvage therapies. Pazopanib was initiated post-transplant, and radiological improvement of residual disease was observed after one year [14].

Given the limitations of our small, retrospective, single-center case series, our observations should be interpreted cautiously. Six pediatric patients with high-risk ES were treated with pazopanib; five achieved sustained complete remission, while one experienced relapse after prolonged stable disease. Detailed information regarding treatment initiation criteria, dose adjustments, laboratory monitoring, and adverse event management was limited and collected retrospectively. Although these findings may suggest a potential clinical benefit in selected patients, the small cohort size and lack of a comparative group limit generalizability.

Treatment was generally well tolerated: all patients experienced hair depigmentation, and only one developed reversible hepatotoxicity requiring dose adjustment. These findings are consistent with previously reported pediatric safety data [11, 18].

To our knowledge, apart from the case reported by Tamura et al. ^14^, this is the first case series describing pazopanib as maintenance therapy in pediatric ES. While our results suggest a possible clinical benefit, larger prospective and controlled studies are required to better define the efficacy, safety, and optimal clinical context for pazopanib use in ES.

## Conclusions

In this retrospective case series, we describe our institutional experience with pazopanib administered as maintenance therapy in high-risk pediatric patients with ES. Pazopanib was generally well tolerated in this cohort. Although several patients achieved prolonged remission, the small sample size and retrospective design limit the strength of any conclusions regarding efficacy.

These findings may support a potential role for pazopanib as a maintenance option in carefully selected patients; however, prospective studies are needed to more clearly define its efficacy, safety, and optimal clinical context in ES.

## Data Availability

No datasets were generated or analysed during the current study.

## Abbreviations

ES: Ewing Sarcoma
PNET: Primitive Neuroectodermal Tumor
VDC/IE: Vincristine, Doxorubicin, Cyclophosphamide / Ifosfamide, Etoposide
VIDE: Vincristine, Ifosfamide, Doxorubicin, Etoposide
IE: Ifosfamide, Etoposide
VAI: Vincristine, Actinomycin D, Ifosfamide
VAC: Vincristine, Actinomycin D, Cyclophosphamide
VIT: Vincristine, Irinotecan, Temozolomide
OS: Osteosarcoma
HDCT: High–Dose Chemotherapy
ASCT: Autologous Stem Cell Transplantation
PFS: Progression–Free Survival
CI: Confidence Interval

## References

[1] Anderson WJ, Doyle LA (2021) Updates from the 2020 world health organization classification of soft tissue and bone tumours. Histopathology 78:644–657 33438273 10.1111/his.14265

[2] Ferrari S, Hall KS, Luksch R, Tienghi A, Wiebe T et al (2011) Nonmetastatic ewing family tumors: high-dose chemotherapy with stem cell rescue in poor responder patients. Results of the Italian sarcoma group/Scandinavian sarcoma group III protocol. Ann Oncol 22(5):1221–1227 21059639 10.1093/annonc/mdq573

[3] Miser SJ, Kralio MD, Tarbell NJ, Pink MP, Fryer CJH et al (2004) Treatment of metastatic ewing’s sarcoma or primitive neuroectodermal tumor of bone: evaluation of combination Ifosfamide and etoposide–a children’s cancer group and pediatric oncology group study. J Clin Oncol 22(14):2873–2876 15254055 10.1200/JCO.2004.01.041

[4] Huang C, Yu QP, Ding Z, Zhou Z, Shi X (2022) The clinical characteristics, novel predictive tool, and risk classification system for primary ewing sarcoma patients that underwent chemotherapy: A large population-based retrospective cohort study. Cancer Med 12(5):6244–6259 36271609 10.1002/cam4.5379 PMC10028057

[5] Paulussen M, Ahrens S, Craft AW, Dunst J, Fröhlich B et al (1998) Ewing’s tumors with primary lung metastases: survival analysis of 114 (European Intergroup) cooperative ewing’s sarcoma studies patients. J Clin Oncol 16(9):3044–3052 9738574 10.1200/JCO.1998.16.9.3044

[6] Spunt SL, McCarville MB, Kun LE, Poquette CA, Cain AM et al (2001) Selective use of whole-lung irradiation for patients with ewing sarcoma family tumors and pulmonary metastases at the time of diagnosis. J Pediatr Hematol Oncol 23(2):93–98 11216713 10.1097/00043426-200102000-00005

[7] Oberlin O, Rey A, Desfachelles AS, Philip T, Plantaz D et al (2006) Impact of high-dose Busulfan plus Melphalan as consolidation in metastatic ewing tumors: a study by the Société Française des cancers de l’enfant. J Clin Oncol 24(24):3997–4002 16921053 10.1200/JCO.2006.05.7059

[8] Ladenstein R, Pötschger U, Le Deley MC, Whelan J, Paulussen M, Oberlin O et al (2010) Primary disseminated multifocal ewing sarcoma: results of the Euro-EWING 99 trial. J Clin Oncol 28(20):3284–3291 20547982 10.1200/JCO.2009.22.9864

[9] Le Deley MC, Paulussen M, Lewis I, Brennan B, Ranft A, Whelan J et al (2014) Cyclophosphamide compared with Ifosfamide in consolidation treatment of Standard-Risk ewing sarcoma: results of the randomized noninferiority Euro-EWING99-R1 trial. J Clin Oncol 32(23):2440–2448 24982464 10.1200/JCO.2013.54.4833

[10] Womer RB, West DC, Krailo MD, Dickman PS, Pawel BR et al (2012) Randomized controlled trial of interval-compressed chemotherapy for the treatment of localized ewing sarcoma: A report from the children’s oncology group. J Clin Oncol 30(33):4148–4154 23091096 10.1200/JCO.2011.41.5703 PMC3494838

[11] Bisogno G, De Salvo GL, Bergeron C, De Salvo GL, Guerin F et al (2019) Vinorelbine and continuous low-dose cyclophosphamide as maintenance chemotherapy inpatients with high‐risk rhabdomyosarcoma (RMS 2005): a multi-centre, open‐label, randomised, phase 3 trial. Lancet Oncol 20(11):1566–1575 31562043 10.1016/S1470-2045(19)30617-5

[12] van der Graaf WT, Blay JY, Chawla SP, Kim DW, Bui-Nguyen B, Casali PG et al (2012) Pazopanib for metastatic soft tissue sarcoma (PALETTE): a randomised, double-blind, placebo-controlled phase 3 trial. Lancet 379(9829):1879–1886 22595799 10.1016/S0140-6736(12)60651-5

[13] Glade Bender JL, Lee A, Reid JM, Baruchel S, Roberts T, Voss SD et al (2013) Phase I Pharmacokinetic and pharmacodynamic study of pazopanib in children with soft tissue sarcoma and other refractory solid tumors: a children’s oncology group phase I consortium report. J Clin Oncol 31:3034–3043 23857966 10.1200/JCO.2012.47.0914 PMC3739862

[14] Tamura A, Yamamoto N, Nino N, Ichikawa T, Nakatani N, Nakamura S et al (2019) Pazopanib maintenance therapy after tandem high-dose chemotherapy for disseminated Ewing sarcoma. Int Canc Conf J 8(3):95–100 10.1007/s13691-019-00362-w PMC6545189 31218182

[15] Seto T, Song MN, Trieu M, Yu J, Sidhu M, Liu CM et al (2019) Real-World experiences with pazopanib in patients with advanced soft tissue and bone sarcoma in Northern California. Med Sci 7(3):48 10.3390/medsci7030048 PMC6473235 30889920

[16] Aggerholm-Pedersen N, Rossen P, Rose H, Safwat A (2020) Pazopanib in the treatment of bone sarcomas: clinical experience. Translational Oncol 13(2):295–299 10.1016/j.tranon.2019.12.001 PMC6931211 31875575

[17] Alcindor T (2015) Response of refractory ewing sarcoma to pazopanib. Acta Oncol 54(7):1063–1064 25345493 10.3109/0284186X.2014.971938

[18] Attia S, Okuno SH, Robinson SI, Webber NP, Indelicato DJ, Jones RL et al (2015) Clinical activity of pazopanib in metastatic extraosseous ewing sarcoma. Rare Tumors 7(2):5992 26266019 10.4081/rt.2015.5992 PMC4508650

[19] Yamamoto Y, Nozawa M, Shimizu N, Minami T, Yoshimura K, Uemura H (2014) Pazopanib for recurrent extraosseous ewing’s sarcoma of the retroperitoneum. Int J Urol 21(11):1183–1184 25040171 10.1111/iju.12546

[20] Brizini M, Naccache L, Tibout P, Goudie C, Budd C, Vairy S et al (2023) Multicenter real-world experience of the clinical efficacy and tolerance of pazopanib in high-risk pediatric solid tumors (PazoPed). Pediatr Hematol Oncol 40(7):643–658 36825687 10.1080/08880018.2023.2182854

[21] Mori Y, Kinoshita S, Kanamori T, Kataoka H, Joh T, Iida S et al (2018) The successful treatment of metastatic extraosseous ewing sarcoma with pazopanib. Intern Med 57(18):2753–2757 29780156 10.2169/internalmedicine.9879-17 PMC6191593

[22] Xu Jie X, Lu, Sun X, Dong S, Tang X (2019) Management of recurrent or refractory ewing sarcoma: A systematic review of phase II clinical trials in the last 15 years. Oncol Lett 18(1):348–358 31289506 10.3892/ol.2019.10328 PMC6540207

[23] Koscielniak E, Sparber-Sauer M, Scheer M, Vokuhl C, Kazanowska B et al (2021) Extraskeletal ewingsarcoma in children, adolescents, and young adults. An analysis ofthree prospective studies of the cooperative Weichteilsarkom-studiengruppe (CWS). Pediatr Blood Cancer 68(10):e29145 34089219 10.1002/pbc.29145

[24] Raciborska A, Bilska K, Rodriguez-Galindo C (2019) Maintenance treat-ment with trofosfamide in patients with primary bone ewing sarcoma—single center experience. Dev Period Med 23:39–44 30954980 10.34763/devperiodmed.20192301.3944 PMC8522343

[25] DuBois SG, Krailo MD, Glade-Bender J, Buxton A, Laack N et al (2023) Randomized phase III trial of ganitumab with Interval-Compressed chemotherapy for patients with newly diagnosed metastatic ewing sarcoma: A report from the children’s oncology group. J Clin Oncol 41(11):2098–2107 36669140 10.1200/JCO.22.01815 PMC10082251

